# High-Content Screening Identifies Vanilloids as a Novel Class of Inhibitors of NET Formation

**DOI:** 10.3389/fimmu.2019.00963

**Published:** 2019-04-30

**Authors:** Elvira Sondo, Roberta Bertelli, Emanuela Pesce, Gian Marco Ghiggeri, Nicoletta Pedemonte

**Affiliations:** ^1^UOC Genetica Medica, IRCCS Istituto Giannina Gaslini, Genova, Italy; ^2^UOC Nefrologia, IRCCS Istituto Giannina Gaslini, Genova, Italy

**Keywords:** NETosis, high-content screening, vanilloids, NETs, inhibitors, neutrophil

## Abstract

Neutrophils migrate to sites of infection where they phagocytose, degranulate, and/or, in the presence of appropriate stimuli, release decondensed chromatin strands (called neutrophil extracellular traps, NETs) for trapping and possibly killing microorganisms. NET formation is characterized by marked morphological cell changes, in particular within the nucleus. Lytic NET formation can be observed in neutrophils undergoing cell death, which is referred to as NETosis. Dysregulation of NET production and/or degradation can exert pathogenic effects, contributing to the pathogenesis of various diseases, including cystic fibrosis, autoimmune diseases and inflammatory conditions. By employing a phenotypic assay based on high-content imaging and analysis, we screened a library of biologically active compounds and identified vanilloids as a novel class of chemical compounds able to hinder NETosis induction and NET release. Vanilloids also markedly decrease cytosolic ROS production. The identification of novel vanilloid NET inhibitors, able to stop excessive or aberrant NET production might offer new therapeutic options for those disorders displaying NET overproduction.

## Introduction

Neutrophil granulocytes are polymorphonuclear cells that migrate to sites of infection (as part of the first line of immune defense) where they phagocytose, degranulate and/or, in the presence of appropriate stimuli, they may undergo marked morphological cell changes, in particular within the nucleus. Indeed, the characteristic nuclear lobules of neutrophils disappear, and neutrophils release NET-like structures of decondensed chromatin strands (called neutrophil extracellular traps, NETs) decorated with antimicrobial peptides, for trapping and possibly killing microorganisms [(1, 2), p. 57]. Lytic NET formation can be observed in neutrophils undergoing cell death, which is referred to as NETosis. Although NET production is involved in host defense and NETs have a potentially protective role against infections, their excessive production or ineffective removal can exert pathogenic effects, resulting in tissue/organ damage, and contribute to the pathogenesis of various diseases (3–5). It has been known for over 40 years that cystic fibrosis patients have high levels of cell-free DNA in their airways (correlating with obstructive lung disease), of which NETs have recently emerged as the principal source (6–8). In addition, antimicrobial peptides coating the NETs are directly cytotoxic to tissue, and can result in deleterious inflammation of host tissue. It has been demonstrated that NETs can directly cause epithelial and endothelial cell death (9).

There is growing evidence that NET formation can exacerbate different diseases in addition to cystic fibrosis. In Systemic Lupus Erythematosus (SLE) and other autoimmune diseases, the abnormal regulation of NETs (with an imbalance between NET formation and clearance) was proposed to play a prominent role in the perpetuation of autoimmunity and disease worsening, i.e., the induction of kidney failure in Lupus Nephritis (10). NETs have also been associated with inflammatory disorders such as arthritis and gout (11, 12). More recently, NETs have been shown to interact with macrophages and to prime inflammation in vascular disease (13). Thus, the identification of drug-like small molecules able to modulate the NET formation pathways could provide clinicians with adjuvant therapies to be used in a patient-specific and disease-dependent fashion. In cystic fibrosis, these drugs could decrease free DNA levels in airways that proved to correlate with obstructive lung disease. In SLE and other autoimmune diseases, modulators of NET formation could inhibit auto-antibody production, triggered by the exposure of post-translational modified proteins in NET remnants, thereby reducing glomerular immune-complex deposition and kidney damage. Indeed, *in vivo* preclinical testing of two PAD inhibitors, Cl-amidine and BB-Cl-amidine, demonstrated that these compounds decreased NET formation and protected against renal, skin and vascular manifestations in murine models of lupus (14, 15). Similarly, Cl-amidine reduced the severity of arthritis in a mouse model of inflammatory arthritis (16).

*In vitro* studies on NET production initially focused on phorbol 12-myristate 13-acetate (PMA), a robust NET inducer that mimics the oxidative burst occurring in inflammation and after microbial infections. PMA activates protein kinase C (PKC), leading to calcium influx, assembly of NADPH oxidase and/or mitochondrial activation, with production of reactive oxygen species (ROS), including hydrogen peroxide (H_2_O_2_), that is consumed by myeloperoxidase (MPO) to produce oxidants (2, 17). ROS production triggers the activation of neutrophil elastase (NE) and its dissociation from the azurosome, a membrane-associated complex of NE, MPO, cathepsin G and other proteins. NE translocates to the nucleus where it cleaves histones and concurs to chromatin decondensation (17). Finally, nuclear envelope and, subsequently, plasma membrane break down, resulting in NET release. A key role in NET formation is also played by gasdermin D (GSDMD), a pore-forming protein that is considered an executor of pyroptosis, a particular cell death that preferentially occurs in monocytes and macrophages. GSDMD, proteolytically activated by NE and other neutrophil proteases, forms pores in the granule membrane, thus enhancing NE release into the cytoplasm and allowing further GSDMD cleavage in a reiterative process (18). In addition, upon completion of NETosis, cleaved GSDMD forms pores in the plasma membrane, allowing NET release (18, 19).

Whereas, NADPH oxidase activation was long considered an absolute requirement for NET release induction, further studies on the molecular mechanisms revealed the existence of alternative pathways that involve protein-arginin deiminase (PAD4) and are strictly calcium-dependent. Indeed, NET formation can be induced experimentally by calcium ionophores or by nigericin, a potassium ionophore (17). These pathways require neither NE nor MPO recruitment, and are independent of cytosolic ROS levels (17). Calcium influx activates PAD4, an enzyme that converts arginine to citrulline on histones, thus weakening the interaction of DNA with histones and promoting chromatin decondensation in the neutrophil nucleus.

Various small molecule-inhibitors of NET formation were reported so far, targeting key molecules or steps of this process, like NADPH oxidase (20), ROS production (21, 22), PKC (23, 24), RAF-MEK-ERK pathway (23), NE (25), MPO (22, 26, 27). In 2017, Martinez and coworkers reported the discovery of tetrahydroisoquinolines acting as inhibitors of NET formation, although their mechanism of action was not clarified (28). Very recently, the first assay to monitor NET formation, based on high-content imaging, was developed and used to screen a small library of 56 compounds (29). Here, we report the development of a novel, optimized phenotypic assay, based on high-content image analysis, to detect pharmacological modulators of NET production, suitable for the screening of large libraries of chemical compounds. This assay relies on an algorithm, developed using machine-learning techniques and signal multiparametric analysis, specifically designed to detect morphological changes associated with NET formation. In particular, our algorithm can recognize lytic NET formation, which we will refer to as NETosis. We employed this assay to screen a library of biologically active compounds, and identified vanilloids as a novel class of chemical compounds able to hinder NETosis induction and NET release.

## Materials and Methods

### Neutrophil Separation and Induction of NET Formation

Human neutrophils were isolated from peripheral blood of healthy volunteers and collected in sodium heparin blood collection tube. Briefly, neutrophils were separated following the Dextran-Ficoll separation protocol and contaminating erythrocytes were lysed by hypotonic shock, as previously described (30). Neutrophils were then resuspended in Hank's Balanced Salt Solution (HBSS) supplemented with 2 mM CaCl_2_ and 0.05% Fetal Calf Serum (FCS), and plated (40,000/well) on polylysine-coated black high-quality 96-well clear-bottom black plates suitable for high-content imaging. Neutrophil suspensions were then incubated at 37°C, 5% CO_2_; after 10 min, NET formation was induced by exposing cells to either Phorbol 12-myristate, 13-acetate (PMA; Sigma P8139) for up to 210 min or ionomycin (Sigma I9657) for up to 90 min.

### Library of Compounds and Library Screening

Master plates of the in-house library of 488 biologically active compounds were originally diluted at 10 mM in DMSO and stocked at −80°C until use. The complete list of compounds included in the library is provided in Supplementary Table 1. Immediately before assays, working plates were prepared at 500 μM by diluting compounds from master plates in PBS. Test compounds were then added to cells at a final concentration of 5 μM in the presence of either 100 nM PMA (for 210 min) or 5 μM ionomycin (for 90 min), unless otherwise indicated.

### High-Content Assay for Visualization of NET Formation

To evaluate NET formation, at the end of treatment neutrophils were formalin-fixed and nucleic acid stain Hoechst 33342 was added to visualize alterations in nuclear morphology and DNA extrusion. Neutrophil plates were then imaged either in non-confocal mode with a 20X air objective, or in confocal mode with a 40X water-immersion objective using the Opera Phenix (PerkinElmer) high-content screening system. Hoechst 33342 signal was laser-excited at 405 nm and the emission wavelengths were collected between 435 and 480 nm.

### Algorithm for Automated Image Analysis

Automated image analysis was performed using the Harmony software of the Opera Phenix high-content screening system, by means of an automated algorithm, developed using machine-learning techniques. This algorithm allows identification of neutrophils, undergoing sequential steps of NETosis, by means of a multiparametric analysis of signal morphology, intensity, density, and texture. In particular, the analysis of signal texture (describing the spatial arrangement of signal intensities in an image) is based on evaluation of Haralick and Gabor features, two subsets of well-known parameters (31, 32), and evaluation of SER (Spots, Edges, Ridges) features, developed by PerkinElmer and included in the Harmony software of Opera Phenix. For our analysis, neutrophils were considered as NETotic only when lytic NET formation was observed.

### Evaluation of Cytosolic ROS Production

To detect intracellular ROS production, neutrophils, exposed to test compounds in combination with 100 nM PMA or 5 μM ionomycin, were stained with Hoechst 33342 to visualize nuclei and with the ROS-sensitive cell-permeant probe 2′,7′-dichlorodihydrofluorescein diacetate (H_2_DCFDA; 1.5 μM). Upon cleavage of the acetate groups by intracellular esterases and oxidation by cytosolic ROS, the non-fluorescent H_2_DCFDA is converted to the highly fluorescent 2′,7′-dichlorofluorescein (DCF). Cytosolic ROS production was then monitored in living neutrophils for up to 120 min by time-lapse imaging of cells using the Opera Phenix high-content screening system. DCF signal was laser-excited at 480 nm and the emission wavelengths were collected between 505 and 535 nm.

### Evaluation of Mitochondrial Viability

The effect of ionomycin on mitochondrial viability was assessed in neutrophils treated with 5 μM ionomycin alone or in combination with test compounds, using tetramethylrhodamine methyl ester (TMRM; 20 nM), a cell-permeant fluorescent dye that is readily sequestered by active mitochondria. NET formation and TMRM accumulation were simultaneously monitored, on living neutrophils, by high-content imaging for up to 60 min after treatment, using the Opera Phenix system. TMRM signal was laser-excited at 560 nm and the emission wavelengths were collected between 570 and 630 nm.

### Evaluation of MPO Expression Following NET Formation

MPO expression was monitored by immunofluorescence in formalin-fixed, non-permeabilized neutrophils under resting conditions or following pharmacological treatment, using an anti-MPO antibody (ab11730 from Abcam) conjugated with phycoerythrin (PE). PE signal was laser-excited at 488 nm and the emission wavelengths were collected between 570 and 630 nm.

### Quantification and Statistical Analysis

Intra-donor variability was assessed by performing three independent experiments (on separate days). Experiments were performed on neutrophils deriving from three different subjects to account for inter-donor biological variability. Thus, for each condition, *n* = 9. The Kolmogorov-Smirnov test was used to evaluate the assumption of normality. Normally distributed data are expressed as means ± SEM. For normally distributed quantitative variables, the one-way parametric analysis of variance (ANOVA) was performed, followed by the Dunnet multiple *post-hoc* test to avoid multiple comparison errors when comparing more than 2 groups.

## Results

**Figure 1:**
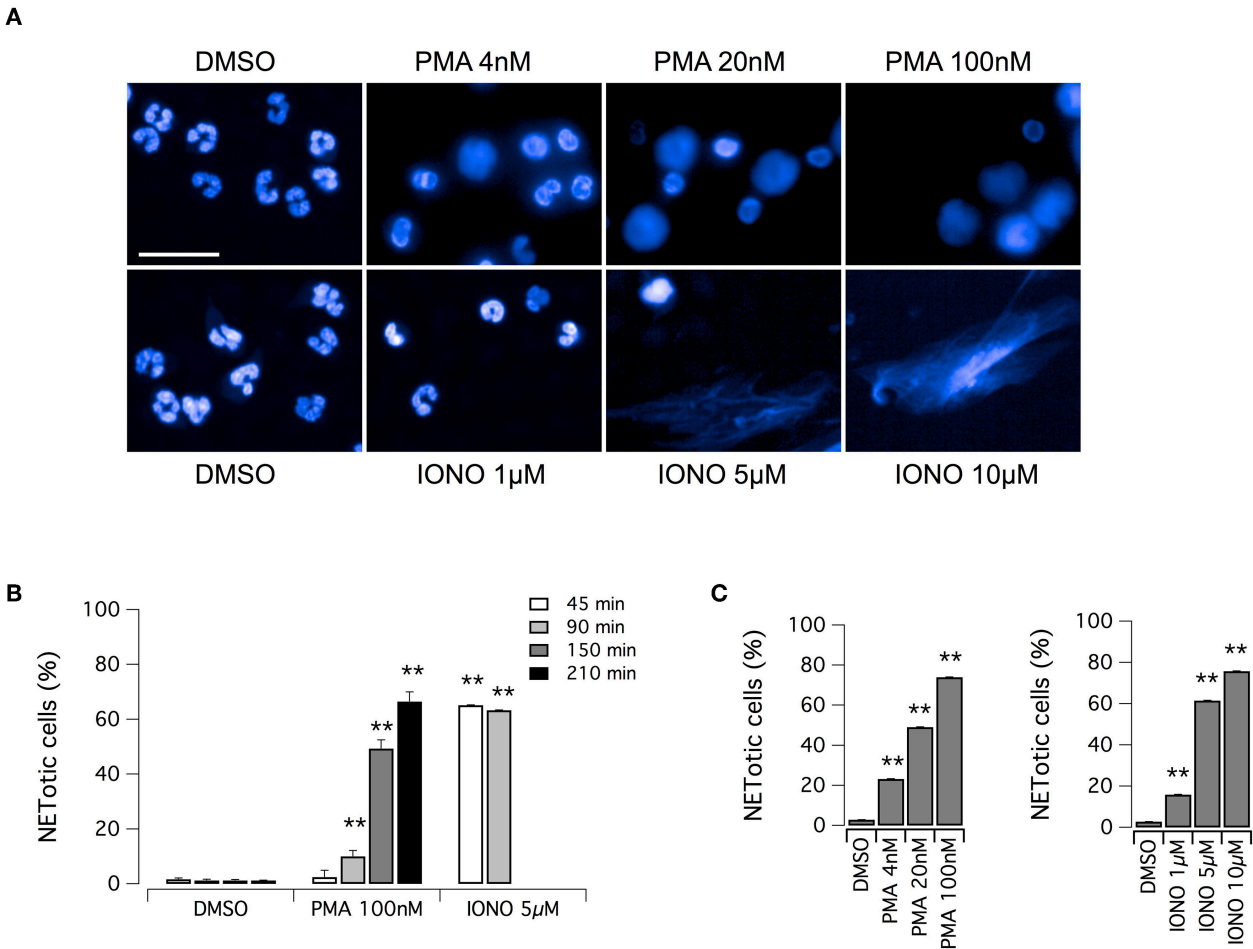
Induction of NET formation in human neutrophils. **(A)** Confocal analysis of morphological changes occurring in Hoechst 33342-stained nuclei of human neutrophils freshly isolated from healthy donor, following treatment with PMA (for 210 min) or ionomycin (for 90 min) or vehicle alone (DMSO), as indicated. Bar = 30 μm. **(B)** Time-dependence of NET production following stimulation with PMA or ionomycin. **(C)** Dose-dependence of netosis induction by PMA (left graph) or ionomycin (right graph). Mean ± SEM, *n* = 9. Asterisks indicate statistical significance vs. the corresponding control (DMSO-treated cells): **p < 0.01.

The aim of this study was the identification of putative modulators of NET formation. We first evaluated the feasibility of Opera Phenix System for the detection and quantification of NETs, through the detailed nuclear morphology analysis in neutrophils exposed to PMA or ionomycin, after formalin fixation and Hoechst 33342 staining. We employed the Harmony software of the Opera Phenix screening system, by means of an automated algorithm, developed using machine-learning techniques, and based on the multiparametric analysis of signal morphology, intensity, density and texture. For each well, and therefore for each experimental condition, we individually analyzed 25,000–30,000 neutrophils. Both PMA and ionomycin induced easily detectable changes in nuclear morphology, with disappearance of the characteristic lobules of the nuclei and extrusion of the chromatin both in the cytoplasm and possibly, in later steps, in the extracellular space, due to the breach of nuclear envelope and plasma membrane (Figure 1A). However, the time-course and the intensity of NET production were dependent on the stimulus applied. Indeed, NET formation was detected in > 60% of neutrophils exposed to ionomycin (5–10 μM) after a short treatment (45 min); conversely, when neutrophils were treated with PMA at the maximal effective dose (100 nM), the same percentage of NETotic cells was observed after 210 min (Figures 1B,C). In addition to this, nuclear alterations induced by 100 nM PMA were less evident than those elicited by ionomycin, that caused a dramatic loss of the nuclear shape with massive extracellular chromatin extrusion (Figure 1A and Supplementary Figure 1). Notably, neutrophils treated with vehicle alone (DMSO) did not undergo any morphological changes in the same exposure time frame, with a percentage of spontaneously NETotic neutrophils lower than 1–2% (Figure 1 and Supplementary Figure 1). To confirm that the chromatin-containing structures detected by Hoechst 33342 staining under these experimental conditions were indeed NETs, we counterstained neutrophils for MPO. MPO expression was detected in both PMA- and ionomycin-induced NETs, although at different levels, being higher in PMA-induced NETs (Supplementary Figure 2). In control neutrophils, treated with vehicle alone (DMSO), no MPO was detected, unless when permeabilized before staining (Supplementary Figure 2). These results suggest that, in cells detected as NETotic by our algorithm, plasma membrane integrity was compromised. In addition, our algorithm of analysis allows discriminating between NETosis and other forms of cell death, like apoptosis, based on their different phenotypic features: indeed, neutrophils treated with etoposide, a known inducer of apoptosis (33), showed reduced nucleus area, but markedly increased Hoechst 33342 signal intensity and different signal texture (Supplementary Figure 2).

**Figure 2:**
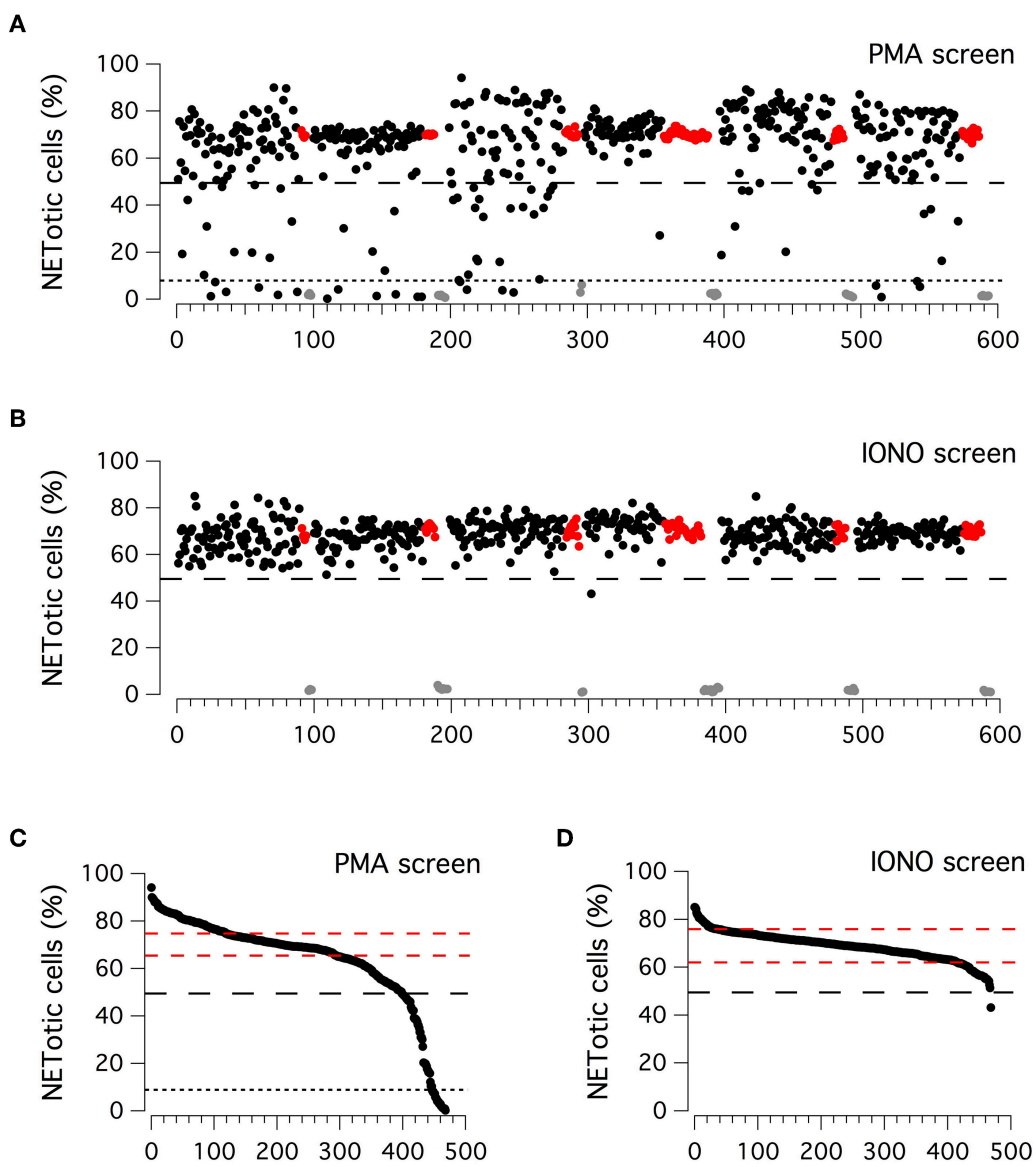
Primary screenings to identify putative inhibitors of NET production. **(A,B)** Results of the compound library screenings performed on freshly isolated human neutrophils, following 210 min treatment with PMA (100 nM; upper panel) or 90 min treatment with ionomycin (5 μM; lower panel). Each dot represents the percentage of NETotic cells in each well, following the different pharmacological treatments. Black dots: cells treated with a single test compound (5μM) in the presence of PMA or ionomycin. Gray dots: cells treated with DMSO alone, in the absence of PMA or ionomycin. Red dots: cells treated with DMSO in the presence of PMA or ionomycin. **(C,D)** Ordered distributions of the PMA **(C)** and ionomycin **(D)** screening displayed in **(A,B)**, respectively. Dashed red lines indicates standard deviation of NETotic cell percentage scores. Dashed and dotted (only in **C**) black lines indicate the thresholds of 50 and 10% NETotic cells, respectively.

Considering the high reproducibility and robustness of this assay, we employed it to screen an in-house library of approximately 500 biologically active compounds, to identify compounds able to inhibit PMA-induced and/or ionomycin-induced NET formation (Figure 2). This library included kinase inhibitors, endocannabinoids, bioactive lipids, modulators of the autophagic pathway and Wnt signaling, and a small set of orphan drugs and FDA-approved drugs. Compounds were added at the concentration of 5 μM to neutrophils stimulated for 210 min with 100 nM PMA or for 90 min with 5 μM ionomycin, after which plates were washed, formalin-fixed, processed to stain nuclei and finally imaged. The ability of compounds to modulate NET production was determined by evaluating the difference in the percentage of NETotic cells as compared to that in the presence of stimuli alone. Results of the two screenings are displayed in Figures 2A,B. The Z' factor (34) was very satisfactory for all screenings: 0.96 for PMA-treated neutrophils and 0.88 for ionomycin-treated neutrophils. The NETotic cell percentage scores were then put into ordered distributions (Figures 2C,D for the screens performed with PMA and ionomycin stimulation, respectively). Since both PMA and ionomycin treatment caused the induction of NETotic phenotype in approximately 70% of the neutrophils, we decided to set a threshold of 30% reduction in NETosis induction rate. Thus, compounds were considered active when the percentage of NETotic neutrophils was below 50%. The primary screening on neutrophils treated with PMA highlighted 70 putative hits, i.e., compounds able to decrease the percentage of NETotic cells to <50% (Figure 2C). Of 70 putative hits, 22 compounds were able to decrease the percentage of NETotic cells to <10% (Figure 2C; see also Supplementary Table 2). Not surprisingly, among the positive hits, there were many protein kinase inhibitors, in particular PKC inhibitors, like Ro31-8220, staurosporine, PKC-412, Sphingosine, Palmitoyl-DL-carnitine, and GF109203X. Our list of hits also included celastrol, already known as an inhibitor of PMA-induced NET formation (35). Interestingly, in the shortlist of the 22 hits there were two analogs belonging to the vanilloid chemical class, i.e. capsaicin and its analog dihydrocapsaicin. These two compounds are known agonists of the transient receptor potential vanilloid type 1 (TRPV1) receptor, an ion channel acting as a key molecule in peripheral nociception, activated by many physical and chemical stimuli such as noxious heat, low extracellular pH, divalent cations as well as animal toxins (36).

Different results were obtained from the primary screening on neutrophils treated with ionomycin, in which only one hit was identified, the bisbiguanide alexidine, which was able to reduce to <50% the number of neutrophils undergoing NETotis (Figure 2D).

**Figure 3:**
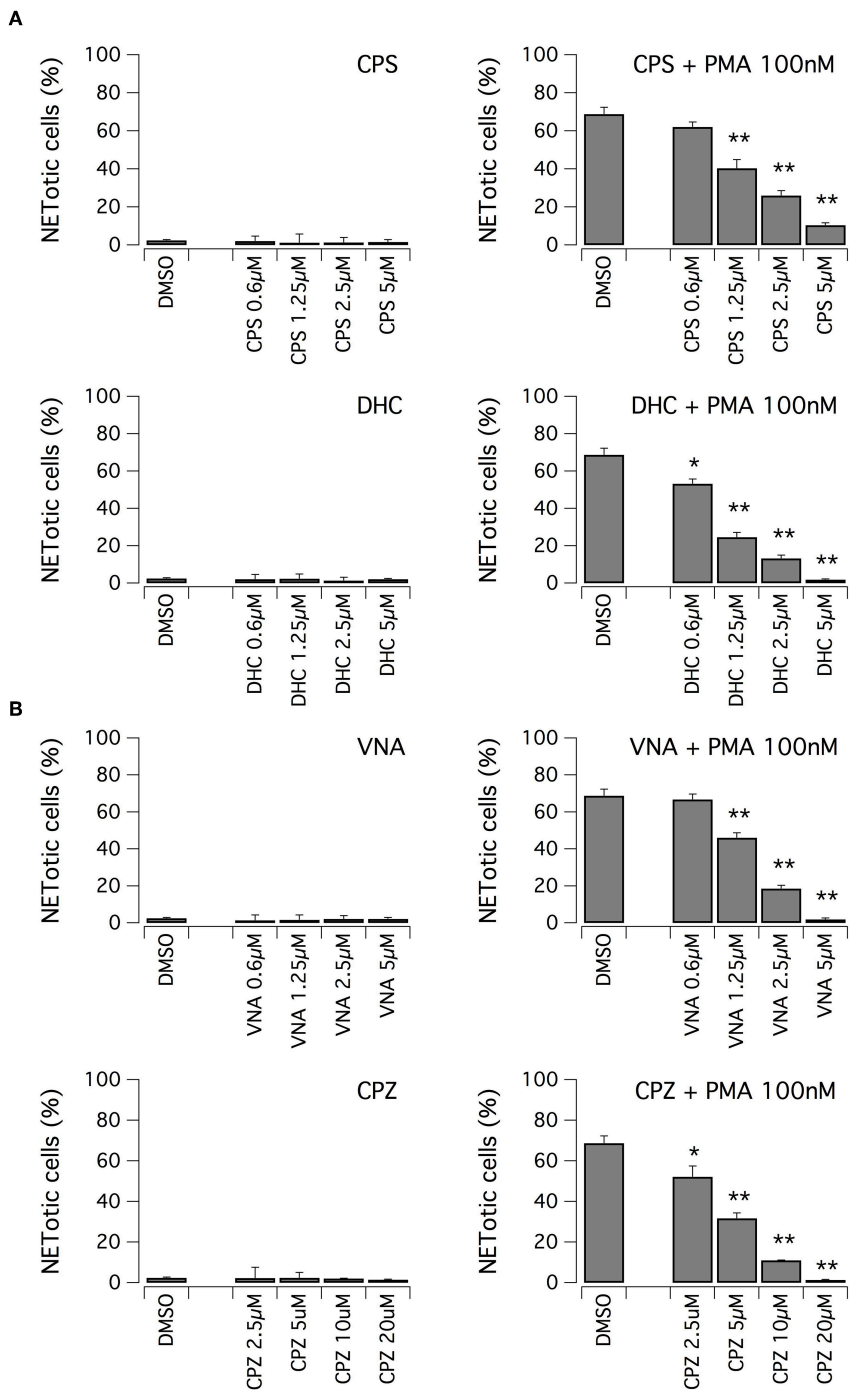
Vanilloids inhibit NET formation by PMA in human neutrophils. **(A)** Dose-response relationships of capsaicin (CPS) and dihydrocapsaicin (DHC) on freshly isolated human neutrophils following 210 min treatment with vehicle alone (DMSO; left panels) or 100 nM PMA (right panels). **(B)** Dose-response relationships of N-vanillylnonanamide (VNA) and capsazepine (CPZ) on freshly isolated human neutrophils following 210 min treatment with vehicle alone (DMSO; left panels) or 100 nM PMA (right panels). Mean ± SEM, *n* = 9. Asterisks indicate statistical significance vs. the corresponding control (DMSO-treated cells): **p < 0.01; *p < 0.05.

We evaluated the ability of hits to inhibit NET formation at different concentrations. First, we focused on vanilloid compounds. The compounds were tested in the low micromolar range on neutrophils in the absence or presence of 100 nM PMA (Figure 3A). Neither capsaicin nor dihydrocapsaicin elicited NET production in neutrophils when the test compounds were applied in the absence of PMA (Figure 3A). Both capsaicin and dihydrocapsaicin were able to inhibit PMA-induced NETosis onset in a dose-dependent manner, with a EC50 in the range of 1–2 μM with complete inhibition achieved at 5 μM (Figure 3A). Besides capsaicin and dihydrocapsaicin, we also took into consideration another two vanilloid structural analogs, N-vanillylnonanamide and capsazepine, that showed similar activity compared to capsaicin and dihydrocapsaicin (Figure 3B).

**Figure 4:**
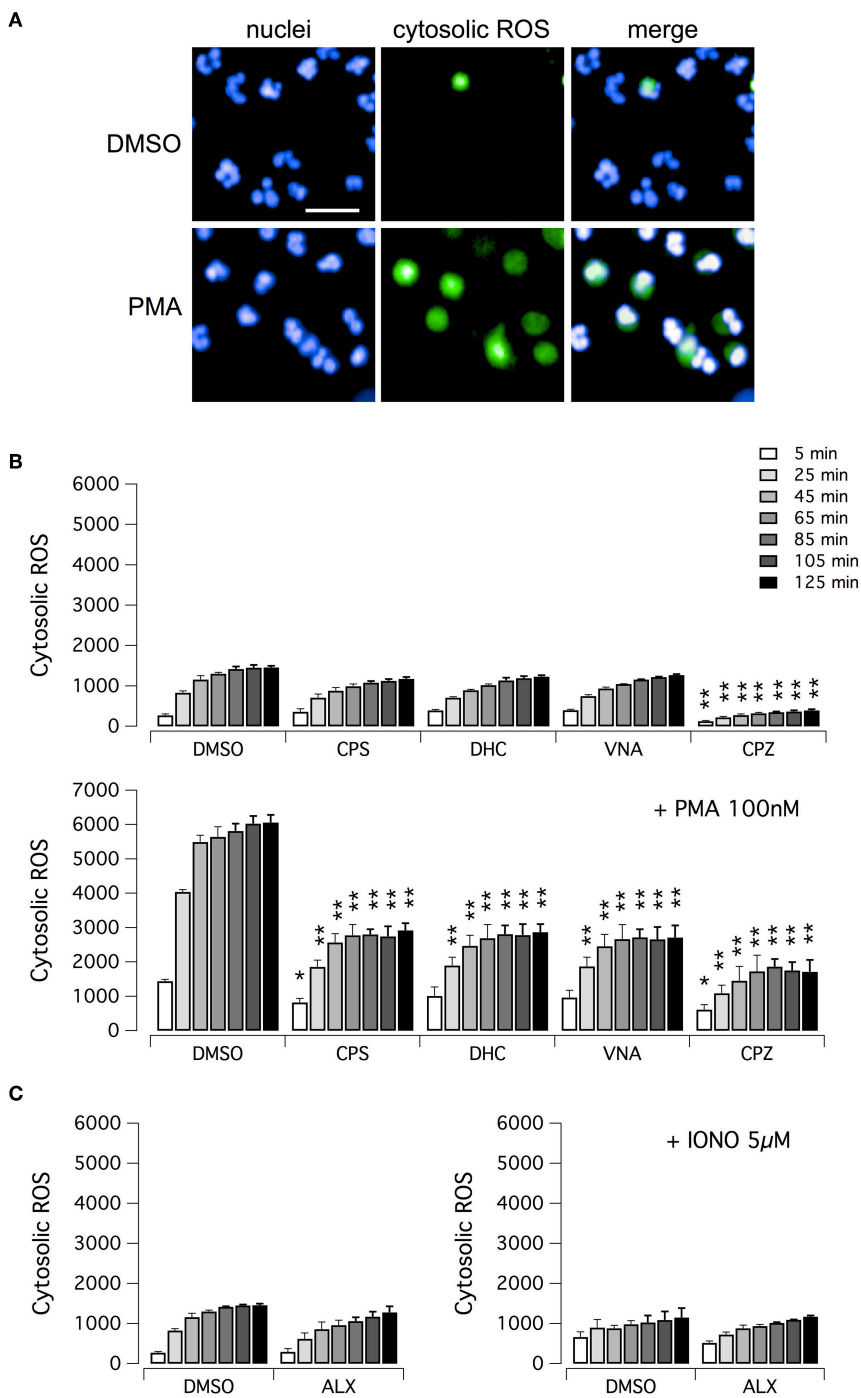
ROS production in human neutrophils. **(A)** Confocal images of human neutrophils freshly isolated from healthy donor, following 120 min treatment with DMSO or 100 nM PMA. Nuclei were stained with Hoechst 33342. Cytosolic ROS were visualized using the ROS-sensitive cell-permeant probe 2′,7′-dichlorodihydrofluorescein diacetate (H_2_DCFDA). Bar = 30 μm. **(B)** Time-dependence of cytosolic ROS production in freshly isolated human neutrophils following treatment with vanilloids (5 μM) in the presence of vehicle alone (DMSO; upper panel) or 100 nM PMA (lower panel). **(C)** Time-dependence of cytosolic ROS production in freshly isolated human neutrophils following treatment with alexidine (5 μM) in the presence of vehicle alone (DMSO; left panel) or 5 μM ionomycin (right panel). Mean ± SEM, *n* = 9. Asterisks indicate statistical significance vs. the corresponding control (DMSO-treated cells): **p < 0.01; *p < 0.05.

We then evaluated the ability of vanilloid compounds to decrease cytosolic ROS following PMA stimulation. To this aim, neutrophils were treated with PMA alone or in combination with test compounds, in the presence of the ROS-sensitive cell-permeant probe 2′,7′-dichlorodihydrofluorescein diacetate (H_2_DCFDA) (Figure 4). Then, NETosis induction as well as cytosolic ROS production were monitored by high-content imaging on living neutrophils for up to 120 min after treatment (Figure 4). In the absence of PMA, the time-dependent increase in H_2_DCFDA fluorescence, proportional to cytosolic ROS production, is quite small, and not influenced by treatment with vanilloids, with the only exception of capsazepine, which caused a significant although modest reduction of cytosolic ROS production (Figures 4A,B). Conversely, in neutrophils stimulated with PMA, H_2_DCFDA fluorescence rose markedly, with a peak of 6-fold increase as compared to non-stimulated neutrophils (Figures 4A,B). Interestingly, the time-course of cytosolic ROS increase reached the steady-state in ~1 h after PMA addition (Figure 4B). As a further control, we also measured cytosolic ROS levels in neutrophils under resting conditions or treated with ionomycin, in the absence or presence of alexidine (Figure 4C). As expected, and in agreement with data in the literature (17), cytosolic ROS were not influenced by these pharmacological maneuvers (Figure 4C).

**Figure 5:**
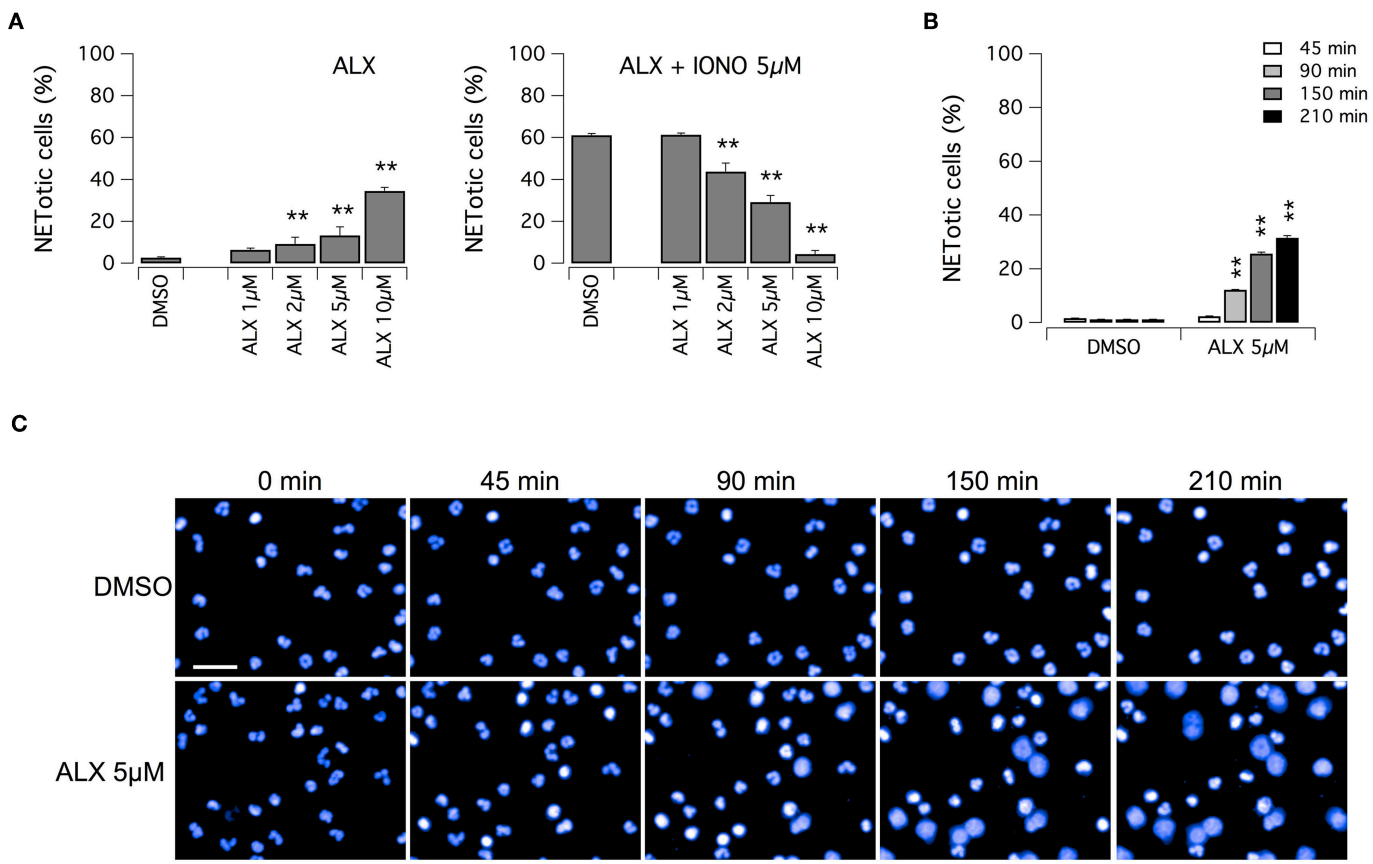
Effect of alexidine treatment on human neutrophils. **(A)** Dose-response relationships of alexidine (ALX) on freshly isolated human neutrophils following 90 min treatment with vehicle alone (DMSO; left panel) or 5 μM ionomycin (right panel). **(B)** Time-dependence of netosis induction by 5 μM alexidine. Mean ± SEM, *n* = 9. Asterisks indicate statistical significance vs. the corresponding control (DMSO-treated cells): **p < 0.01. **(C)** Confocal analysis of morphological changes occurring in Hoechst 33342-stained nuclei of human neutrophils following treatment with 5 μM alexidine. Bar = 30 μm.

Subsequently, we evaluated the ability of alexidine to inhibit NETosis induction by ionomycin. Alexidine was therefore tested at different concentrations in the low micromolar range on unstimulated neutrophils or in the presence of ionomycin (Figure 5A). Interestingly, we observed that alexidine was able to induce NET appearance even in the absence of ionomycin stimulation. On the other hand, with similar dose-response relationship, this compound also inhibited NET production induced by ionomycin (Figure 5A). Thus, we focused our attention on the ability of alexidine to elicit NETosis. We evaluated the time-course of NET formation induced by alexidine (Figures 5B,C). Interestingly, a small fraction of neutrophils treated with a submaximal concentration of alexidine (5 μM) began to display NETotic morphological features already after 90 min of treatment, however the maximal effect was achieved in 150–210 min (Figures 5B,C). MPO expression was detected in alexidine-induced NETs, although at lower levels as compared to PMA-induced NETs (Supplementary Figure 3).

**Figure 6:**
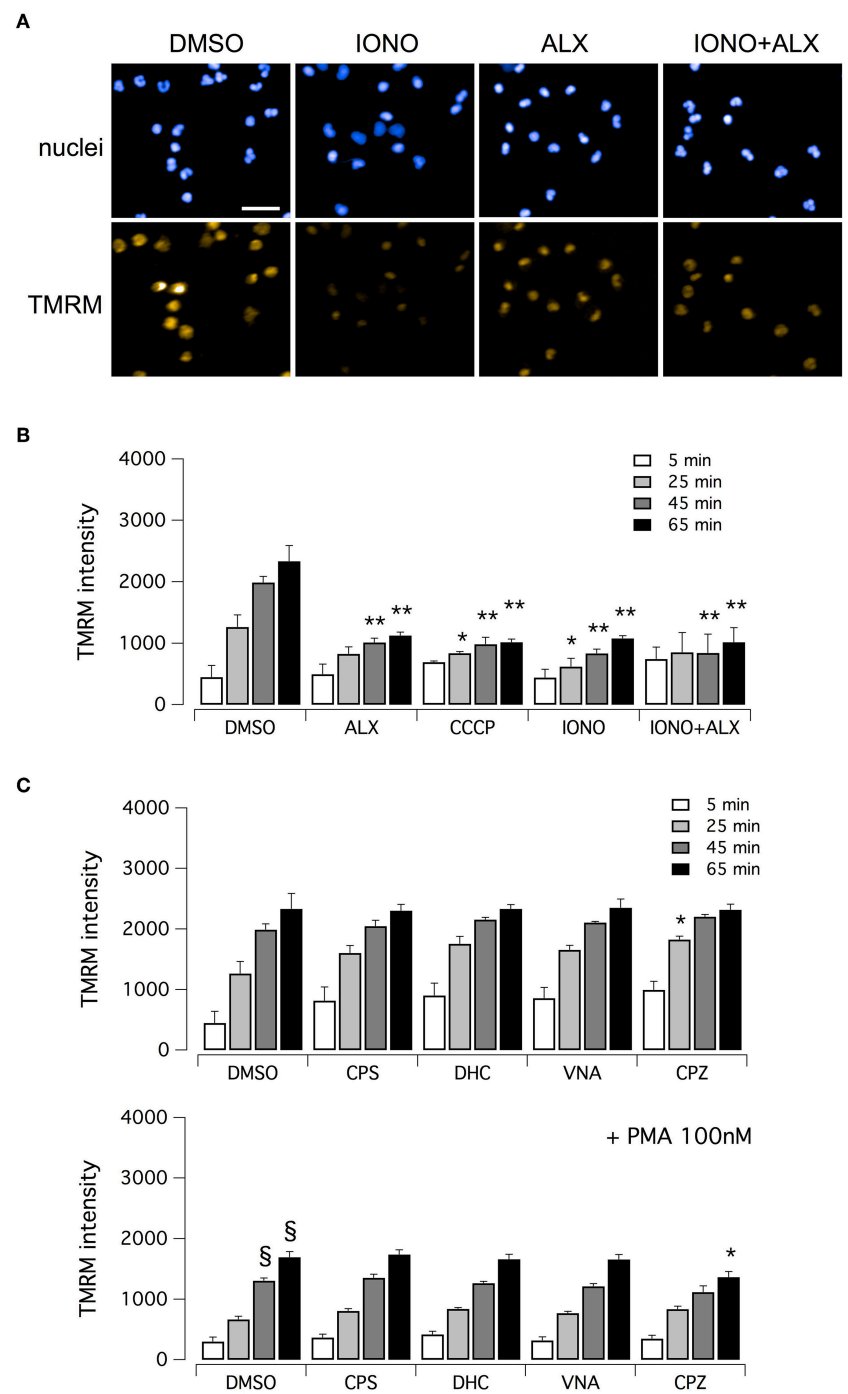
Evaluation of mitochondrial viability in human neutrophils. **(A)** Confocal images of human neutrophils freshly isolated from healthy donor, following 60 min treatment with DMSO or 5 μM ionomycin in the absence or in the presence of 5 μM alexidine. Nuclei were stained with Hoechst 33342. Mitochondria were visualized with tetramethylrhodamine methyl ester (TMRM), a cell-permeant fluorescent dye that is readily sequestered by active mitochondria. Bar = 30 μm. **(B)** Time-dependence of TMRM accumulation in freshly isolated human neutrophils following treatment with 5 μM alexidine in the presence of DMSO or 5 μM ionomycin. The uncoupling agent carbonyl cyanide m-chlorophenyl hydrazone (CCCP), a chemical inhibitor of oxidative phosphorylation, was used as a positive control for mitochondrial impairment. **(C)** Time-dependence of TMRM accumulation in freshly isolated human neutrophils following treatment with 5 μM vanilloids. Mean ± SEM, *n* = 9. Symbols indicate statistical significance vs. the corresponding control (DMSO-treated cells): **p < 0.01; *p < 0.05; §*p* < 0.05 (vs. DMSO treated cells in the absence of PMA stimulation, shown in **C**, top graph).

Finally, we evaluated mitochondrial viability following neutrophil treatment with ionomycin. Indeed, calcium ionophores proved to elicit the generation of a large amount of mitochondrial ROS (37). It has been demonstrated that oxidative stresses caused by mitochondrial ROS increase can induce rapid depolarization of mitochondrial membrane potential (38). Thus, we assessed mitochondrial viability in neutrophils treated with ionomycin alone or in combination with test compounds, and in the presence of tetramethylrhodamine methyl ester (TMRM), a cell-permeant fluorescent dye that is readily sequestered by active mitochondria (Figure 6). TMRM accumulation was monitored, in parallel to NET formation, for up to 60 min after treatment by high-content imaging on living neutrophils (Figure 6). In the absence of ionomycin stimulation, TMRM was accumulated in active mitochondria, leading to a time-dependent increase in TMRM fluorescent signal in the mitochondria (Figures 6A,B). The protonophore, carbonyl cyanide m-chlorophenylhydrazone (CCCP), that uncouples mitochondria, induced a marked reduction in TMRM accumulation (Figure 6B). A similar reduction in TMRM accumulation was also observed when neutrophils were treated with ionomycin, or alexidine or ionomycin plus alexidine (Figure 6B). As a further control, we evaluated the effect of vanilloids on mitochondria viability. Interestingly, vanilloid compounds did not alter either the extent or the time-dependence of TMRM accumulation (Figure 6C). Stimulation with PMA slightly slowed down TMRM accumulation, an effect that was not prevented by co-treatment with vanilloids (Figure 6C).

## Discussion

A pathogenic role for NET formation has been postulated, if not demonstrated, for various diseases, including SLE and other autoimmune diseases, inflammatory conditions like arthritis, gout and vasculitis, and possibly also for cystic fibrosis (6, 7, 10–13). Thus, there is an increasing interest in the identification of drug-like small molecules able to slow down or inhibit NET release, which could represent attractive therapeutic tools. Indeed, in the last few years various assays have been developed and optimized to screen for modulators of NET production, based on different readout methodologies and suitable for different throughputs. For example, Martinez and colleagues (28) developed a phenotypic assay in which neutrophils were imaged using an automated microscope and analyzed using image analysis software. The screening of an in-house library of 8407 natural product-inspired compounds led to the identification of a group of substituted tetrahydroisoquinolines as a class of small molecules that inhibit PMA-induced NET formation at micromolar concentrations (28). Similarly, Chicca and colleagues developed and optimized a phenotypic assay based on high-content imaging and analysis that was used to screen a small panel of 56 compounds including a wide range of pharmaceutically relevant compounds with varied targets for immune cell modulation (29). In this article, the authors stressed the main advantage deriving from the use of high-content analysis, that is the potential to analyze a large number of neutrophils per sample, rather than only a small fraction. In addition, these authors highlighted the importance of sample handling and processing: considering that NETs are fragile structures and that their integrity is essential for accurate analysis, the fixative procedures were optimized to guarantee sample preservation (29). Thus, we focused our attention on sample preparation first. We opted for a different strategy from that adopted by Chicca et al. (29). We found that polylysine coating improved neutrophils attachment to the plastic plates, without altering the response to pharmacological NET-inducers and preserving the morphological changes that are peculiar of NETotis onset. In addition, polylysine coating did not interfere with either non-confocal or confocal high-content imaging protocols required for our studies. The effectiveness of our protocols for sample handling and processing was demonstrated by the extremely low occurrence (~1%) of neutrophils undergoing spontaneous cell death (through either NETosis or apoptosis).

Using the Harmony software of the Opera Phenix system and machine-learning techniques, we developed an algorithm specifically designed for image analysis of morphological changes associated with NETotis. This algorithm can perform a multiparametric analysis based on analysis of signal morphology, intensity, density, and texture (i.e., the spatial arrangement of signal intensities), thus allowing the identification of neutrophils undergoing sequential steps of NETosis, up to complete disruption of the nuclear envelope and eventually loss of plasma membrane integrity (lytic NET formation). Indeed, it has been reported that NET formation could occur also in the absence of NETosis, hence in a sort of “reversible” condition (39, 40). Thus, for our studies, neutrophils were considered as NETotic only when complete disruption of the nuclear envelope was observed, as evidenced by chromatin filling up the whole cytoplasm. In addition, our algorithm of analysis allows discriminating between NETosis and other forms of cell death, like apoptosis, having different phenotypic features, without the need of specific markers.

This assay, taking advantage of high-content imaging and analysis procedures, allowed us to individually analyze 25,000–30,000 neutrophils for each experimental condition, resulting in great statistical power.

We screened a set of targeted libraries, i.e., collections of compounds having known biological activities and targeting known molecular effectors of specific cellular pathways. These libraries are useful to dissect the molecular pathways involved in a yet uncharacterized biological phenomenon, and thus to identify novel drug targets. The screens were designed to identify negative modulators of NETosis induction following treatment with either PMA or ionomycin. The robustness of our assay was testified by the Z' factor calculated for the screenings: 0.96 for PMA-treated neutrophils and 0.88 for ionomycin-treated neutrophils.

Our screens identified 70 putative inhibitors of PMA-induced NETosis, 22 of which able to fully inhibit NETosis onset. Among the hits, we identified several kinase inhibitors, consistently with data in the literature (23, 24). Interestingly, the screen also highlighted two vanilloids as compounds able to hinder PMA-induced NETosis, i.e., capsaicin and dihydrocapsaicin. Interestingly, these compounds displayed higher potency as compared to previously identified inhibitors of NET formation like tetrahydroisoquinolines, since complete inhibition was achieved at 5 μM, while tetrahydroisoquinolines were maximally effective at 30 μM (28). Capsaicin and dihydrocapsaicin are known agonists of the TRPV1 receptor (36). However, TRPV1 involvement in NET formation and release could be excluded since we found that also capsazepine, a known antagonist of TRPV1 receptors (41), was able to arrest NET production by PMA, preventing the increase in cytosolic ROS. It has been reported that capsaicin and capsazepine can suppress nuclear factor κB (NFκB) activation (42) and that celastrol, an inhibitor of NET formation (also identified in our screening), acts possibly through downregulation of the SYK-MEK-ERK-NFκB signaling cascade (35). Thus, most likely also vanilloids act similarly.

Our studies also highlighted the differences occurring between the mechanisms of NETosis induction elicited by PMA and ionomycin, as already described (17). Interestingly, our screen identified only one compound able to stop ionomycin-induced NETosis, the bisbiguanide alexidine, which was also a NETosis inducer, although with a slower time-course. Alexidine is an antiseptic agent used in mouthwashes to eliminate plaque-forming microorganisms. It binds to lipopolysaccharide and lipoteichoic acid and inhibits fungal phospholipase B (43). In addition, alexidine also inhibits the mitochondrial protein tyrosine phosphatase PTP localized to the Mitochondrion 1 (PTPMT1) (44). By inhibiting mitochondrial phosphatase PTPMT1 alexidine causes hyperactivation of succinate dehydrogenase (SDH), an enzyme that participates in both the Krebs cycle and in the mitochondrial respiratory complex II of the electron transport chain (45). It has been reported that mitochondrial complex II can generate reactive oxygen species at high rates in both the forward and reverse reactions, thus suggesting that complex II may be an important contributor to physiological and pathological ROS production (46). Thus, although alexidine can inhibit NET formation elicited by ionomycin, we hypothesize that it can also induce NETosis possibly by increasing mitochondrial ROS.

In summary, our studies identified a novel class of inhibitors of PMA-induced NETosis, the vanilloids. Capsaicin has been utilized to treat many diseases, with particular focus on pain (47). Capsaicin patches are safe and proved to be effective in controlling neuropathic pain resulting from post herpetic neuralgia, post-surgical neuralgia, post-traumatic neuropathy, polyneuropathy, and mixed pain syndrome (47). In addition, potential use of capsaicin in the upper airways has been proposed, as adjuvant therapy for both allergic and non-allergic rhinitis (47). Capsaicin has also been evaluated as treatment for burning mouth syndrome, resulting in long-lasting but partial symptom relief, although associated with important collateral effects (47).

The identification of novel NET inhibitors, able to stop excessive or aberrant NET production might offer new therapeutic options for those disorders displaying NET overproduction like cystic fibrosis, SLE, and inflammatory diseases (3–7). Indeed, preclinical studies on murine models have already demonstrated the therapeutic relevance of PAD inhibitors (14–16). In addition, capsaicin and other vanilloids prevented the production of cytosolic ROS, suggesting that these compounds may be useful to limit the hyper-responsiveness of neutrophils observed under certain pathological conditions like periodontitis (48).

## Ethics Statement

This study was approved by the Regional Ethical Committee (Comitato Etico Regionale) on October 24, 2014, under the supervision of the Italian Ministry of Health. Written informed consent was obtained from all donors using a form that was also approved by the same Ethical Committee.

## Author Contributions

ES, RB, and NP conceptualization. ES, EP, and RB investigation and validation. ES, RB, and NP writing. GMG and NP funding acquisition. GMG and NP resources.

### Conflict of Interest Statement

The authors declare that the research was conducted in the absence of any commercial or financial relationships that could be construed as a potential conflict of interest.

## Supplementary Material

Supplementary Figure 1 Induction of NET formation in human neutrophils. Confocal analysis of morphological changes occurring in Hoechst 33342-stained nuclei of human neutrophils freshly isolated from healthy donor, following treatment with PMA (for 210 min) or ionomycin (for 90 min) or vehicle alone (DMSO), as indicated. White squares highlight the regions shown in Figure 1A. The Bar = 100 μm.

Supplementary Figure 2 MPO expression during NET formation. A and B. Confocal analysis of MPO expression and morphological changes occurring in Hoechst 33342-stained nuclei of human neutrophils following treatment with PMA (100 nM for 210 min) or ionomycin (5 μM for 90 min) or etoposide (to induce apoptosis; 25 μM for 210 min) or vehicle alone (DMSO), as indicated. Arrow indicates apoptotic nuclei, identified by the algorithm according to reduced nucleus area, but markedly increased Hoechst 33342 signal intensity and different signal texture. Bar = 40 μm for images in **(A)**. Bar = 15 μm for images in **(B)**. Images in **(B)** were taken with longer exposure times.

Supplementary Figure 3 MPO expression during NET formation. Confocal analysis of MPO expression and morphological changes occurring in Hoechst 33342-stained nuclei of human neutrophils following 210 min treatment with PMA (100 nM) or alexidine (5 μM) or vehicle alone (DMSO), as indicated. Bar = 15 μm.

Supplementary Table 1 Library of biologically-active compounds.

Supplementary Table 2 List of putative hits from the primary screening in PMA-induced neutrophils.
