# Supplementary figures and images for: High-Content Screening Identifies Vanilloids as a Novel Class of Inhibitors of NET Formation
Source: Front Immunol. 2019 Apr 30;10:963. doi: 10.3389/fimmu.2019.00963 (PMC6503056; doi:10.3389/fimmu.2019.00963)

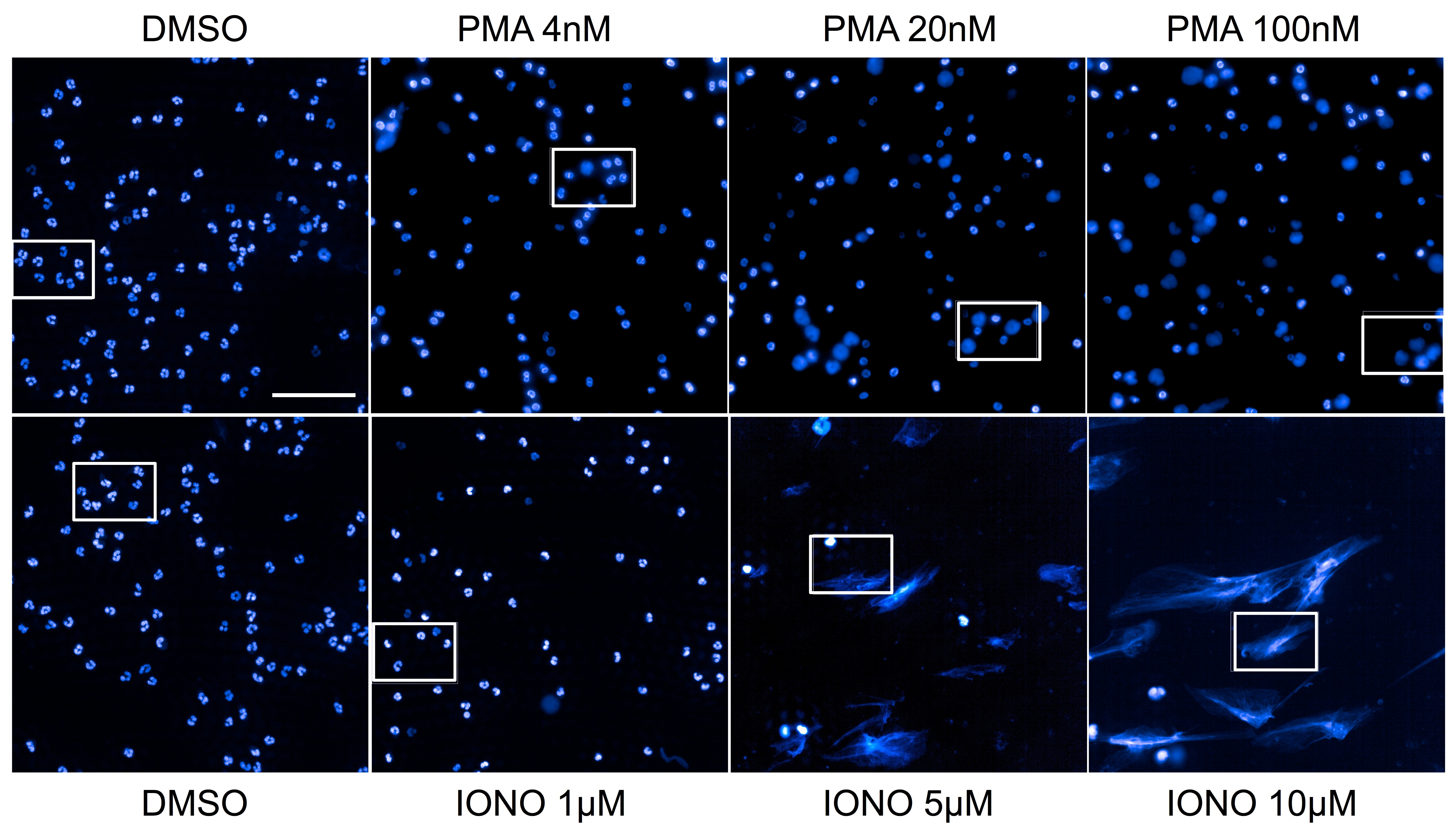

Supplement: Supplementary Figure 1 — Induction of NET formation in human neutrophils. Confocal analysis of morphological changes occurring in Hoechst 33342-stained nuclei of human neutrophils freshly isolated from healthy donor, following treatment with PMA (for 210 min) or ionomycin (for 90 min) or vehicle alone (DMSO), as indicated. White squares highlight the regions shown in Figure 1A. The Bar = 100 μm. [file Image_1.JPEG]

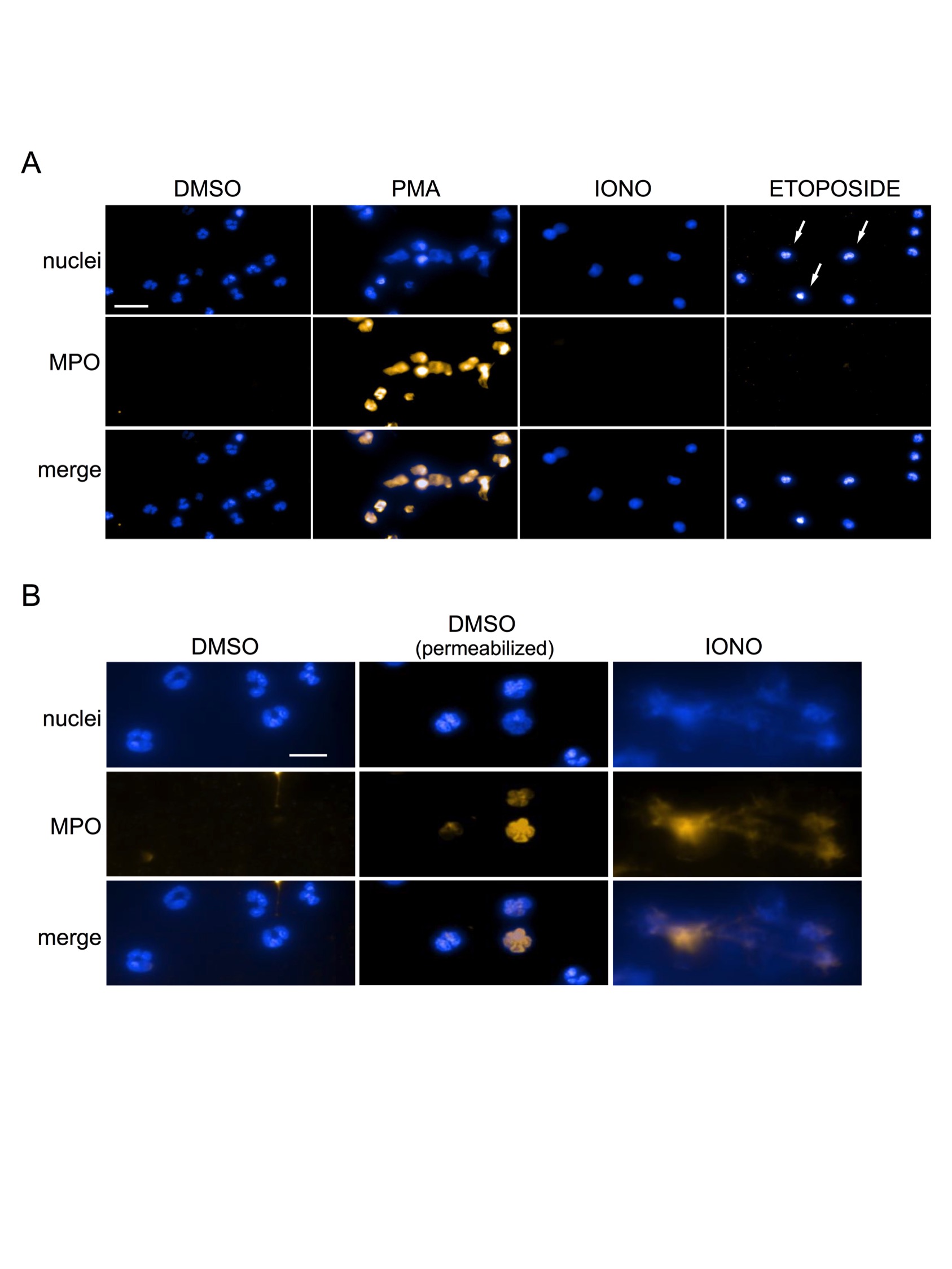

Supplement: Supplementary Figure 2 — MPO expression during NET formation. A and B. Confocal analysis of MPO expression and morphological changes occurring in Hoechst 33342-stained nuclei of human neutrophils following treatment with PMA (100 nM for 210 min) or ionomycin (5 μM for 90 min) or etoposide (to induce apoptosis; 25 μM for 210 min) or vehicle alone (DMSO), as indicated. Arrow indicates apoptotic nuclei, identified by the algorithm according to reduced nucleus area, but markedly increased Hoechst 33342 signal intensity and different signal texture. Bar = 40 μm for images in (A). Bar = 15 μm for images in (B). Images in (B) were taken with longer exposure times. [file Image_2.JPEG]

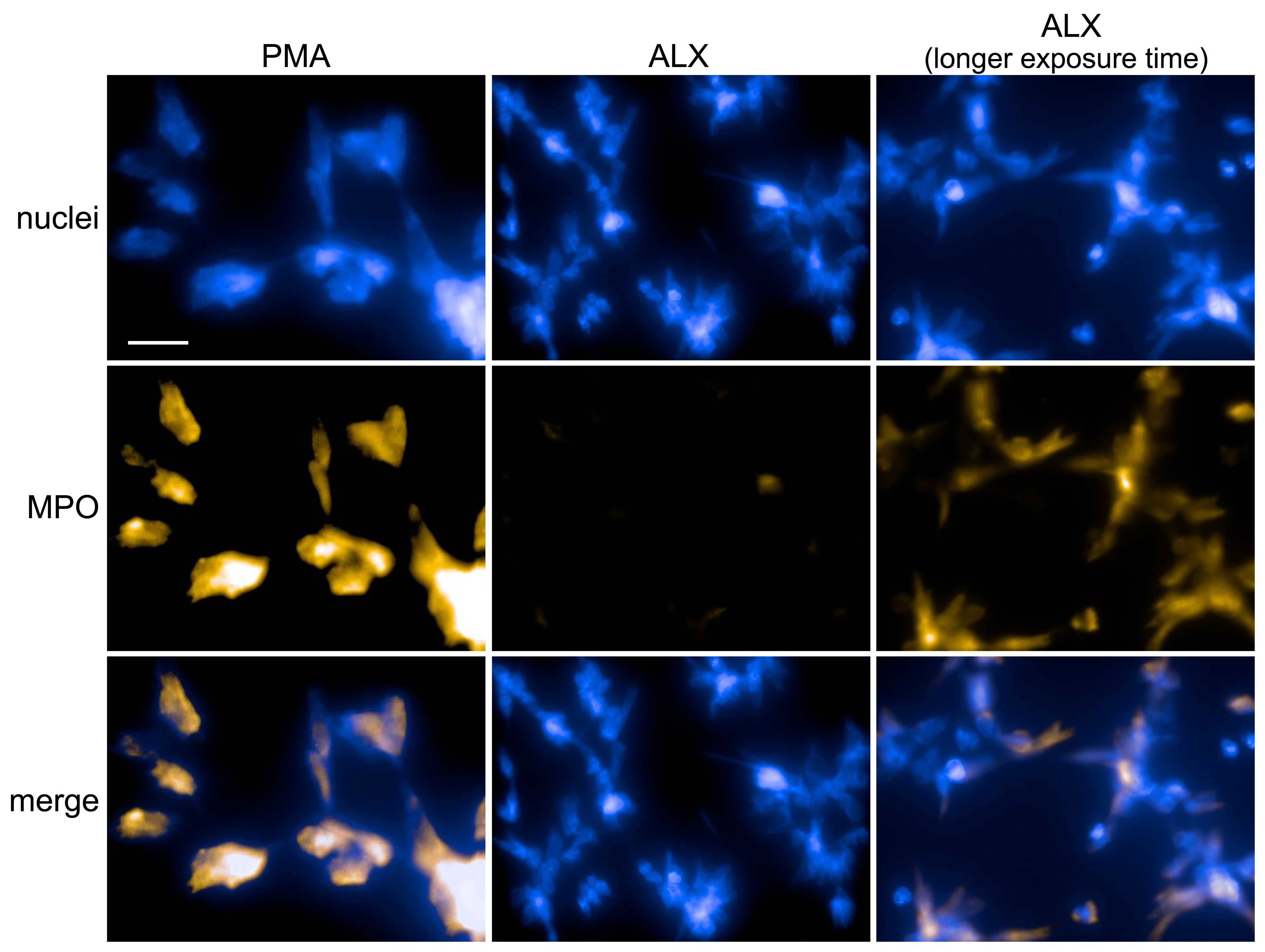

Supplement: Supplementary Figure 3 — MPO expression during NET formation. Confocal analysis of MPO expression and morphological changes occurring in Hoechst 33342-stained nuclei of human neutrophils following 210 min treatment with PMA (100 nM) or alexidine (5 μM) or vehicle alone (DMSO), as indicated. Bar = 15 μm. [file Image_3.JPEG]
