# Supplementary material for: High-Content Screening Identifies Vanilloids as a Novel Class of Inhibitors of NET Formation
Source: Front Immunol. 2019 Apr 30;10:963. doi: 10.3389/fimmu.2019.00963 (PMC6503056; doi:10.3389/fimmu.2019.00963)
Supplement: Supplementary Table 1 — Library of biologically-active compounds. [file Table_1.DOCX]

**Supplementary Table 1. Library of biologically-active compounds.**

| (-)-Nicotine |
| --- |
| (-)3,4-Dihydroxynorephedrine |
| (+)-Nicotine |
| (±)Bay K8644 |
| (1'S,2'S)-Nicotine-1'-Oxide |
| (R,S)-3-(2-Piperidinyl) Pyridine |
| 1,1'-Ethylidene-Bis(L-Tryptophan) |
| 1,2 Dimethyl-3-Hydroxypyrid-4-One |
| 17-AAG |
| 17-Allylamino |
| 2-Aminopurine |
| 2-Deoxyglucose |
| 2-Phenethylamine |
| 2,4-Dihydroxyphenylacetyl-L-Asparagine |
| 2',5'-Dideoxyadenosine |
| 3-Hydroxy-4-Methoxyphenethylamine |
| 3-Hydroxyphenethylamine |
| 3-Methoxytyramine |
| 3-Methyladenine |
| 3,4-Dihydroxyphenylacetic Acid |
| 3253-5986 |
| 4-Hydroxy-3-Methoxy-Phenylacetic Acid |
| 5-Fluoro-1-Uracil |
| 5-Aza-2-Deoxycytidine |
| 5-Fluorocytosine |
| 5-Hydroxy-L-Tryptophan |
| 5-Iodo-2-Deoxyuridine |
| 5-Iodotubercidin |
| 5-Methoxy-DL-Tryptophan |
| 5-Methoxy-N,N-Dimethyltryptamine |
| 5-Methoxytryptophol |
| 5,6-Dichloro-1-B-D-Ribofuranosylbenzimidazole |
| 6-Formylindolo [3,2-B] Carbazole |
| 6-Gingerol |
| 6-Methoxytryptamine |
| 7-Ketocholesterol |
| 8-Azaguanine |
| 8-CPT-cAMP |
| A23187 |
| Abacavir |
| ABC294640áHCl |
| Acarbose |
| Aceclofenac |
| Acenocoumarol |
| Acetamidofenil D-glucoronide |
| Acetohexamide |
| Acitretin |
| Adrenochrome |
| Adrenochrome semicarbazone |
| AG-126 |
| AG-1296 |
| AG-370 |
| AG-490 |
| AG-494 |
| AG-825 |
| AG-879 |
| AG112 |
| Agmatine sulfate |
| AICAR |
| Akt Inhibitor X |
| Alendronate |
| Alexidine |
| Alfuzosin |
| ALLN |
| Alvespimycin |
| Amcinonide |
| Aminosalicylic acid |
| Amiodarone |
| Amlexanox |
| Amoxapine |
| Amprenavir |
| Anagrelide |
| Anandamide |
| Anastrozole |
| Anisomycin |
| Antimycin |
| Apigenin |
| AR-42 |
| AR-A014418 |
| AS605240 |
| Astemizole |
| Atazanavir |
| Atracurium |
| Aufanofin |
| Azithromycin |
| Aztreonam |
| Bafilomycin A1 |
| Bambuterol |
| BAY 11-7082 |
| Belinostat |
| Benzthiazide |
| Benzydamine |
| Betaxolol |
| Bexarotene |
| Bicalutamide |
| BIO |
| BML-257 |
| BML-259 |
| BML-265 |
| BML-284 |
| BML285 |
| BML286 |
| Boldine |
| Bortezomib |
| Bosentan |
| Bosutinib |
| Box5 |
| Brefeldin A |
| Bromocriptine |
| Bromperidol |
| Budesonide |
| Buflomedil |
| Bumetanide |
| Butoconazole |
| C2-dihydroceramide |
| Caffeine |
| Calcipotriene |
| Calmidazolium |
| Capecitabine |
| Capsaicin |
| Carbachol |
| Carbamazepine |
| Carbamyl-β-Methylcholine |
| Cardamonin |
| Carnosol |
| CCT036477 |
| Cefadroxil |
| Cefdinir |
| Cefditoren |
| Cefotaxime |
| Cefotetan |
| Cefpodoxime |
| Ceftibuten |
| Celastrol |
| Celecoxib |
| Cephaeline |
| Cetirizine |
| Cevimeline |
| Chetomin |
| CHIR99021 |
| Chloroquine |
| Chlorothiazide |
| Chlortetracycline |
| Chlorzoxanone |
| Cladribine |
| Clavulanate |
| Clobetasone |
| Clonidine |
| Cotinine |
| Curcumin |
| Cycloheximide |
| Cyproheptadine |
| cyproterone |
| Cytidine 5'-diphosphocholine |
| D,L-Nornicotine |
| D4476 |
| Daidzein |
| DAPT |
| Daptomycin |
| Darifenacin |
| Deflazacort |
| Dehydroisoandrosterone |
| Delavirdine |
| Deoxycholic acid |
| Dequalinium |
| Desipramine |
| Desloratidine |
| Desmopressin |
| Desogestrel |
| Desoximetasone |
| DHA |
| Diarylsulfonesulfonamide |
| Dibromo-L-tyrosine |
| Dibutyryl cAMP |
| Diclofenac |
| Didanosine |
| Diflorasone |
| Digitoxigenin |
| Dihydrocapsaicin |
| Dihydroergocristine |
| DimeflinE |
| Dinoprostone |
| Diperodon |
| Disulfiram |
| DL-4-Hydroxy-3-methoxy-mandelic acid |
| Dofetilide |
| Doxazosin |
| Doxorubicin |
| Doxylamine |
| Drospirenone |
| DTT |
| Duloxetine |
| Dutasteride |
| Dyanocobalamin |
| Dydrogesterone |
| Ebastine |
| Efavirenz |
| EGCG |
| EHNA |
| ETA |
| Ethyl β-carboline-3-carboxylate |
| Etidronate |
| Etoposide |
| Exifone |
| Ezetimibe |
| FG-4592 |
| FH-535 |
| FK-866 |
| Flavanone |
| Fluorometholone |
| Flupenthixol |
| Fluspirilene |
| Fluticasone |
| Forskolin |
| Foxy-5 |
| Gallic acid |
| Ganciclovir |
| GDC-0941 |
| Gedunin |
| Gefitinib |
| Geldanamycin |
| Gemcitabine |
| Genistein |
| Gentamycin |
| GF 109203X |
| Glafenine |
| Glipizide |
| Glucosamine HCl |
| Go6850 |
| Gossypol |
| Granisetron |
| GW 5074 |
| GW9662 |
| H-7 |
| H-8 |
| H-89 |
| H-9 |
| HA-1004 |
| HA-1077 |
| Harmaline |
| Harmane |
| Harmine |
| HBDDE |
| HDBA |
| Hexachlorophene |
| HNMPA |
| hydrocortisone |
| Hydroxychloroquine |
| Hypericin |
| Hypotaurine |
| ICG-001 |
| Idoxuridine |
| IM-12 |
| Imatinib mesylate |
| Imidazole-4-acetic acid |
| Imipenem |
| Imiquimod |
| Imperatorin |
| Indigo |
| Indinavir |
| Indirubin |
| Indirubin-3'-monoxime |
| Iohexol |
| iso-Olomoucine |
| Isocarboxazid |
| Isoxicam |
| IWP-2 |
| IWR-1 |
| JS-K |
| Kenpaullone |
| KN-62 |
| KN-93 |
| KU0063794 |
| L-690,330 |
| L-Kynurenine |
| Lamivudine |
| Lanatoside C |
| Lansoprazole |
| Lavendustin A |
| Leflunomide |
| Levobunolol |
| LFM-A13 |
| Licochalcone A |
| Loperamide |
| Lopinavir |
| Loratidine |
| Losartan |
| Loteprednol |
| LY294002 |
| LY456236 |
| Mefloquine |
| Menadione |
| Metamizole |
| Metformin |
| Methacycline |
| Methylbenzethonium |
| meticrane |
| MG132 |
| Mifepristone |
| Miglitol |
| Minoxidil |
| Mirtazapine |
| Misoprostol |
| MK 886 |
| ML-7 |
| ML-9 |
| MLN 2238 |
| MLN 9708 |
| MLN4924 |
| Modafinil |
| Moexipril |
| Mometasone |
| Montelukast |
| N-Acetyl-5-Hydroxytrptamine |
| N-Butyl-Β-Carboline-3-Carboxylate |
| N-Methylanabasine |
| N'-Nitrosonornicotine |
| N9-Isopropyl-Olomoucine |
| Naratriptan |
| NCI16221 |
| NDGA |
| Nebivolol |
| Nefazodone |
| Nelfinavir |
| Nevirapine |
| Niclosamide |
| Nicotyrine |
| Nifenazone |
| Nifuratel |
| Niguldipine |
| Nilutamide |
| Nimodipine |
| Nitazoxanide |
| Nitrendipine |
| Nitrofural |
| Nizatidine |
| NO-ASA |
| Nocodazole |
| Norclomipramine |
| NVP-BEZ235 |
| O-Phospho-L-serine |
| Octopamine |
| Oxamic acid |
| Oxybutynin |
| p-Methoxyphenylethylamine |
| Palmitoyl-DL-carnitine |
| Pamidronate |
| Pararosaniline |
| Parthenolide |
| PD-98059 |
| Pemetrexed |
| Penitrem A |
| Permethrin |
| PGE2 |
| Phenacetin |
| Phenazone |
| Phendione |
| Phenoxybenzamine |
| Phosphocreatine |
| PI-103 |
| Piceatannol |
| Pifithrin-µ |
| Pimozide |
| Piracetam |
| Piribedil |
| Pizotifen |
| PKC-412 |
| Plumbagin |
| PMSF |
| PNU-74654 |
| PP1 |
| PP2 |
| Pramipexole |
| Pregabalin |
| Primaquine |
| Progesterone |
| Proparacaine |
| Proscillaridin |
| Pterostilbene |
| Purpurogallin |
| Pyridoxine |
| Pyrvinium pamoate |
| QS-11 |
| Quercetin |
| Repaglinide |
| Reserpine |
| Resveratrol |
| Retinoic Acid |
| RG-14620 |
| Ricinine |
| Ro 31-8220 |
| Rockout |
| Rolipram |
| Roscovitine |
| Rosiglitazone |
| Rosuvastatin |
| Rotenone |
| Rottlerin |
| Roxythromycin |
| Rufloxacin |
| S-Sulfo-L-cysteine |
| Salsolinol-1-carboxylic acid |
| Saquinavir |
| SB-202190 |
| SB-203580 |
| SB-216763 |
| SC-514 |
| Scopolamine |
| Securinine |
| Silybin |
| Simvastatin |
| SMER28 |
| Sodium valproate |
| Solifenacin |
| Sorafenib tosylate |
| SP600125 |
| Spaglumic acid |
| Spermidine |
| Sphingosine |
| Spiramycin |
| Spironolactone |
| Staurosporine |
| Stavudine |
| STF-62247 |
| SU 4312 |
| SU11652 |
| SU1498 |
| Sucralfate |
| Sulfaguanidine |
| Sulprostone |
| Tamoxifen citrate |
| Tamsulosin |
| Taurine |
| Tenofovir |
| Thiamine |
| Thioridazine |
| Thiostrepton |
| Tiagabine |
| Ticlopidine |
| Timosaponin A-III |
| Trandolapril |
| Trans-4-Hydroxycrotonic acid |
| Trazodone |
| Trehalose |
| Trichostatin A |
| Triciribine |
| Trifluoperazine |
| Triflusal |
| Trimebutine |
| Troglitazone |
| Tropanylindole |
| Tropisetron |
| Troxerutina |
| TTFA |
| Tunicamycin |
| TWS-119 |
| Tyramine |
| Tyrphostin 1 |
| Tyrphostin 23 |
| Tyrphostin 25 |
| Tyrphostin 46 |
| Tyrphostin 47 |
| Tyrphostin 51 |
| Tyrphostin 9 |
| Tyrphostin AG 1288 |
| Tyrphostin AG 1295 |
| Tyrphostin AG 1478 |
| U-0126 |
| Usnic acid |
| Verapamil |
| Verteporfin |
| Viminol |
| Vindesine |
| Vitamin A |
| Vitamin E |
| Vitamin K1 |
| Voriconazole |
| WAY-262611 |
| Wortmannin |
| WZ 3146 |
| XAV939 |
| Xibornol |
| Y-27632 |
| Z36 |
| Zalcitabine |
| Ziprasidone |
| ZM 336372 |
| ZM 449829 |
