# Supplementary material for: High-Content Screening Identifies Vanilloids as a Novel Class of Inhibitors of NET Formation
Source: Front Immunol. 2019 Apr 30;10:963. doi: 10.3389/fimmu.2019.00963 (PMC6503056; doi:10.3389/fimmu.2019.00963)
Supplement: Supplementary Table 2 — List of putative hits from the primary screening in PMA-induced neutrophils. [file Table_2.DOCX]

**Supplementary Table 2. List of putative hits from the primary screening in PMA-induced neutrophils.**

| Compound | NETotic cells (%) |
| --- | --- |
| (-)3,4-Dihydroxynorephedrine | 18.8378 |
| (±)Bay K8644 | 49.0756 |
| 2',5'-Dideoxyadenosine | 19.2888 |
| 3-Methoxytyramine | 30.9684 |
| 5-Methoxy-N,N-dimethyltryptamine | 46.2302 |
| Adrenochrome | 46.024 |
| Alvespimycin | 49.0383 |
| Anisomycin | 42.2762 |
| BAY 11-7082 | 0.249962 |
| Betaxolol | 43.1516 |
| BIO | 8.1422 |
| Boldine | 7.44996 |
| Caffeine | 48.2257 |
| Capsaicin | 9.3701 |
| CCT036477 | 5.85818 |
| Celastrol | 4.00737 |
| Cephaeline | 9.4433 |
| Chloroquine | 31.0125 |
| Curcumin | 1.26186 |
| D-Asparagine | 49.4729 |
| Dequalinium | 47.4121 |
| Desipramine | 38.8946 |
| Dihydrocapsaicin | 7.2706 |
| Disulfiram | 17.1479 |
| Doxazosin | 16.2957 |
| DTT | 49.7123 |
| Dydrogesterone | 42.6113 |
| EHNA | 20.2313 |
| Exifone | 50.8349 |
| Forskolin | 47.7689 |
| Gedunin | 34.9961 |
| Geldanamycin | 49.3421 |
| GF 109203X | 4.13874 |
| Go6850 | 3.13291 |
| GSK inhibitor | 48.3749 |
| H-89 | 30.159 |
| Imatinib mesylate | 49.5478 |
| Imperatorin | 49.2481 |
| JS-K | 7.66979 |
| Licochalcone A | 20.0893 |
| Loratidine | 15.8515 |
| LY456236 | 5.38503 |
| Menadione | 3.82736 |
| MLN4924 | 38.5922 |
| NDGA | 2.89279 |
| NO-ASA | 36.2881 |
| NVP-BEZ235 | 19.843 |
| Oxamic acid | 39.1957 |
| Palmitoyl-DL-carnitine Cl | 20.3441 |
| PD-98059 | 48.8875 |
| Phenacetin | 46.6529 |
| Phendione | 36.0946 |
| PI-103 | 48.5763 |
| PKC-412 | 1.35676 |
| Plumbagin | 4.98928 |
| Primaquine | 8.5164 |
| Purpurogallin | 38.268 |
| Quercetin | 47.7945 |
| Repaglinide | 38.8434 |
| Ro 31-8220 | 9.2281 |
| Rosiglitazone | 43.7853 |
| Salsolinol-1-carboxylic acid | 46.408 |
| SB-216763 | 16.3639 |
| SB202190 | 17.6673 |
| Scopolamine | 46.3353 |
| Sphingosine | 37.473 |
| Spiramycin | 48.1738 |
| Staurosporine | 2.08908 |
| SU11652 | 47.1189 |
| TTFA | 33.0814 |
| WAY-262611 | 33.2138 |
| Wortmannin | 1.01035 |
| WZ 3146 | 27.1639 |
| Z36 | 49.0614 |
| ZM 449829 | 0.998306 |
